# Fast Adaptive Temperature-Based Re-Optimization Strategies for On-Line Hot Spot Suppression during Locoregional Hyperthermia

**DOI:** 10.3390/cancers14010133

**Published:** 2021-12-28

**Authors:** H. Petra Kok, Johannes Crezee

**Affiliations:** Department of Radiation Oncology, Amsterdam UMC, University of Amsterdam, Cancer Center Amsterdam, 1105 AZ Amsterdam, The Netherlands; h.crezee@amsterdamumc.nl

**Keywords:** hyperthermia, hyperthermia treatment planning, adaptive planning, temperature optimization

## Abstract

**Simple Summary:**

When treatment limiting hot spots occur during locoregional hyperthermia (i.e., heating tumors to 40–44 °C for ~1 h), system settings are adjusted based on experience. In this study, we developed and evaluated treatment planning with temperature-based re-optimization and compared the predicted effectiveness to clinically applied protocol/experience-based steering. Re-optimization times were typically ~10 s; sufficiently fast for on-line use. Effective hot spot suppression was predicted, while maintaining adequate tumor heating. Inducing new hot spots was avoided. Temperature-based re-optimization to suppress treatment limiting hot spots seemed feasible to match the effectiveness of long-term clinical experience and will be further evaluated in a clinical setting. When numerical algorithms are proven to match long-term experience, the overall treatment quality within hyperthermia centers can significantly improve. Implementing these strategies would then imply that treatments become less dependent on the experience of the center/operator.

**Abstract:**

Background: Experience-based adjustments in phase-amplitude settings are applied to suppress treatment limiting hot spots that occur during locoregional hyperthermia for pelvic tumors. Treatment planning could help to further optimize treatments. The aim of this research was to develop temperature-based re-optimization strategies and compare the predicted effectiveness with clinically applied protocol/experience-based steering. Methods: This study evaluated 22 hot spot suppressions in 16 cervical cancer patients (mean age 67 ± 13 year). As a first step, all potential hot spot locations were represented by a spherical region, with a user-specified diameter. For fast and robust calculations, the hot spot temperature was represented by a user-specified percentage of the voxels with the largest heating potential (HPP). Re-optimization maximized tumor T90, with constraints to suppress the hot spot and avoid any significant increase in other regions. Potential hot spot region diameter and HPP were varied and objective functions with and without penalty terms to prevent and minimize temperature increase at other potential hot spot locations were evaluated. Predicted effectiveness was compared with clinically applied steering results. Results: All strategies showed effective hot spot suppression, without affecting tumor temperatures, similar to clinical steering. To avoid the risk of inducing new hot spots, HPP should not exceed 10%. Adding a penalty term to the objective function to minimize the temperature increase at other potential hot spot locations was most effective. Re-optimization times were typically ~10 s. Conclusion: Fast on-line re-optimization to suppress treatment limiting hot spots seems feasible to match effectiveness of ~30 years clinical experience and will be further evaluated in a clinical setting.

## 1. Introduction

Mild hyperthermia treatments, i.e., heating tumors to 40–44 °C, enhance the effect of radiotherapy and chemotherapy [1,2,3,4,5,6,7]. Treatment outcome depends on both the achieved temperature and treatment duration [8,9,10,11,12]. Therefore, the thermal dose is often expressed as the number of equivalent minutes at 43 °C [13]. The standard treatment duration is typically ~1 h to ensure both a good therapeutic effect and patient tolerance. The risk of thermal toxicity to normal tissue also depends on the local temperature and exposure time [14]. In clinical practice, hyperthermia-associated toxicity is rarely observed when temperatures remain below the pain sensation level (~45 °C [15]). The maximum achievable tumor temperature is usually limited when such treatment limiting hot spots occur.

Tumor temperatures should be monitored to ensure adequate heating. Standard clinical thermometry feedback uses (minimally invasive) thermometry probes. For some specific tumor sites (e.g., soft tissue sarcoma or tumors in extremities), non-invasive MR-thermometry can be utilized [16,17], however for patients with thoracic, abdominal or pelvic tumors, the reliability is limited due to organ movement and blood flow [18]. Therefore, quality assurance guidelines [19] prescribe treatment guidance based on thermometry probe information and patient feedback about the incidence of hot spots.

Locoregional hyperthermia is usually applied for heating pelvic tumors, administered by means of phased-array systems, consisting of four or more antennas organized in one or more rings around the patient [20]. Commercially available systems are the BSD-2000 systems and the ALBA-4D system, operating between 60 and 120 MHz [21,22,23,24]. The phases and amplitudes of the antennas can be adjusted such that interference yields a heating focus at the tumor location. However, due to inhomogeneities in dielectric and thermal tissue properties, treatment limiting hot spots can occur at tissue interfaces [25]. The operator should determine phase-amplitude settings to maximize tumor heating, while avoiding treatment limiting normal tissue hot spots. Phase-amplitude steering is presently usually based on experience of the operator and empirical steering protocols.

Hyperthermia treatment planning can improve treatment quality [26,27]. Ideally, inverse treatment planning, i.e., ‘inverting’ the optimal specific absorption rate (SAR)/temperature distribution to obtain phase-amplitude settings that generate that optimal distribution, as also used for radiotherapy, would be applied. A numerical optimization routine would then be used to prescribe optimal phases and amplitudes. Many research groups have worked on such methods, either based on SAR [28,29,30,31,32,33] or temperature [25,31,34,35]. However, due to the uncertainties in predicted SAR and temperature levels, caused by the lack of information about patient-specific tissue properties and local blood perfusion, treatment limiting hot spots can still occur when applying such optimized phase-amplitude settings and on-line adjustments during treatment remain necessary [36,37].

Thus, inverse pre-treatment optimization methods cannot provide a quantitatively reliable and robust treatment plan. However, previous studies have shown qualitative reliability, i.e., when adjusting phase-amplitude settings, the resulting simulated and measured changes in heating patterns do correlate [38,39]. The deviation between measured and predicted changes in temperature after phase-amplitude steering was accurate within ±0.1 °C for most events [39]. This makes adaptive planning during treatment possible. Hyperthermia treatment planning can be very useful in assisting phase-amplitude steering in response to hot spots and to optimize tumor heating [39,40,41]. Visualizing the predicted effect of different steering strategies can help the operator to find more effective phase-amplitude steering strategies [40,41]. In analogy with radiotherapy, this can be considered as ‘forward adaptive planning’.

Inverse adaptive planning strategies based on SAR have been proposed by Canters et al. [42]. In case of a hot spot, the power density in the hot spot region was reduced by adding a penalty term to the objective function. Target temperatures in the therapeutic range were achieved in patients using this complaint-adaptive steering strategy [42]. A cross-over trial with 36 patients further confirmed feasibility of SAR-based complaint-adaptive steering, yet also revealed some challenges in hot spot suppression [43].

Although SAR and temperature are correlated [39], temperature-based treatment planning might be more effective for complaint-adaptive steering strategies. SAR-based treatment planning does not account for the influence of relevant thermal processes as conduction, blood perfusion and water bolus cooling, which makes that SAR hot spots will not always coincide with temperature hot spots. Furthermore, SAR-based treatment planning strategies, as described above, account for hot spots using a penalty term, but do not use normal tissue constraints to suppress and/or avoid hot spots. Temperature-based optimization maximizes the target temperature with constraints to normal tissue temperatures [25]. This makes temperature-based optimization more intuitive to use and potentially more effective to suppress hot spots. However, on-line re-optimization needs to be fast, preferably a few seconds, and at least less than 1 min. A drawback of temperature-based optimization is that it is computationally more expensive than SAR-based optimization, despite efficient computation strategies that have been developed [34,44].

In this study we developed fast temperature-based re-optimization strategies for on-line use, and we evaluated the possible effectiveness of these strategies for 22 hot spots in 16 cervical cancer patients using simulations. Results for re-optimized phase-amplitude settings were compared to measured/predicted results of experience/protocol-based clinically applied steering in terms of hot spot reduction and target temperatures.

## 2. Materials and Methods

### 2.1. Treatment Planning Workflow

Hyperthermia treatment planning is part of the standard clinical workflow at the Amsterdam UMC. First a 60 cm long CT scan is made in the treatment position, i.e., on a water bolus and mattresses, with the thermometry catheters in situ. The radiation oncologist delineates the target region and this standard DICOM structure set and the CT data set are imported by Plan2Heat, a versatile in-house developed finite difference-based software package for hyperthermia treatment planning [45]. Hyperthermia treatment planning is performed by a physicist. Further tissue segmentation is based on Hounsfield Units, distinguishing muscle, fat, bone and lung/air. This process also segments the plastic thermometry catheters as bone, which is corrected manually, along with other segmentation artefacts, if present.

This segmented anatomy is downscaled to 2.5 × 2.5 × 2.5 mm^3^ and combined with a hyperthermia applicator model; in our case the 70 MHz ALBA-4D system with 4 waveguides (top, bottom, left, right). The literature-based dielectric and thermal tissue properties are assigned, with (tissue-dependent) enhanced perfusion values accounting for a physiological response to a temperature rise in the hyperthermic range [46,47,48,49,50]. All voxels labelled in the same tissue category are assigned homogeneous properties. Table 1 summarizes these properties. Using this patient-applicator model, electromagnetic field, SAR and temperature simulations are performed.

### 2.2. Potential Hot Spots

**Figure 1:**
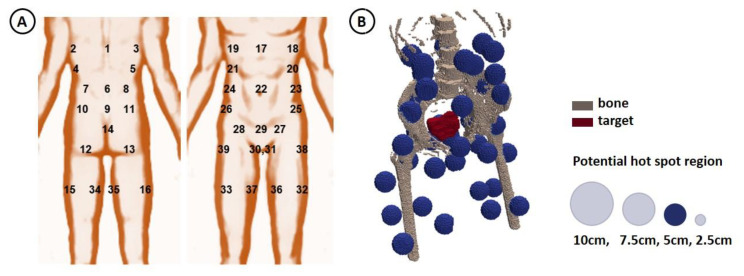
(**A**) Anatomical picture with all potential hot spot locations identified by a unique number, as used during clinical hyperthermia treatments. (**B**) 3D illustration of the spherical regions representing the potential hot spot locations, together with the bony anatomy for a cervical cancer patient. In this study, the potential hot spot diameter was varied between 2.5 and 10 cm. In this visualization a potential hot spot diameter of 5 cm is shown.

At Amsterdam UMC hot spot complaints occurring during clinical hyperthermia treatments are registered in the treatment report. To facilitate this process each potential hot spot location is identified by a unique number (1–39), projected onto an anatomical picture (Figure 1A). This helps communication between the patient and the operator and to assess the hot spot location corresponding to the present pain complaint.

In the treatment planning process, these potential hot spot locations were represented by spherical regions, with a user-specified diameter. For a semi-automatic generation of potential hot spot regions, a dedicated module was added to Plan2Heat, in which the user should specify the desired diameter and eight world coordinates. The first six coordinates represent the center of the spherical region for the potential hot spot locations at the pubic bone (29), tail bone (9), left and right hip (25 + 26), left and right belly (23 + 24). The other two coordinates represent the center of the upper legs at the end of the water bolus, which are used in combination with the six hot spots to approximate the other 33 potential hot spot regions by interpolation. At these coordinates, spherical potential hot spot regions are created. If desired, the user can manually correct specific hot spot locations. A 3D example of reconstructed potential hot spot locations is shown in Figure 1B.

### 2.3. Temperature-Based Re-Optimization

Thermal predictions were based on the commonly applied Pennes’ bioheat equation, where perfusion is modelled as a heat sink [52]. The steady-state temperature *T* (°C) was defined as the steady state solution to:

(1) $$c\rho \frac{\partial T}{\partial t}=\nabla \cdot \left(k\nabla T\right)-c_{b}W_{b}\left(T-T_{art}\right)+\rho \cdot \mathrm{SAR}$$

with *c_b_* (J kg^−1^ °C^−1^) the specific heat capacity of blood (~3600 J kg^−1^ °C^−1^) and *T_art_* the local arterial or body core temperature (assumed to be 37 °C). For efficient calculations during temperature-based optimization, the temperature at voxel (*x,y,z*) was calculated using superposition by:

(2) $$T\left(x,y,z\right)=v^{H}\underline{T}v+T_{00}\;$$

where *v* is the feed vector containing the amplitudes and phases, *T* is a complex *N × N* Hermitian matrix (*N*: number of antennas) and *T*_00_ is a constant representing the thermal boundary conditions. For more details on derivation of these matrix elements, the reader is referred to previously published articles on this topic [25,34]. Quadratic programming was used to optimize a specified objective function, subject to normal tissue and antenna constraints. This was conducted five times with different random initial phase-amplitude settings, after which the best overall result was selected. Objectives and constraints are defined in the subsections below.

#### 2.3.1. Constraints

Normal tissue temperature evaluation is one of the most time consuming operations during constrained temperature-based optimization due to the very large number of normal tissue voxels. Therefore, normal tissue evaluation is reduced to the 39 potential hot spot regions, which are represented by 39 normal tissue constraints. Averaging the hot spot temperature in these regions allows extremely fast calculation, since an average temperature matrix can be used. However, this would largely smooth peak temperatures and thus not be effective in hot spot suppression. Continuously searching for the maximum temperature per hot spot region during optimization is again time consuming and, thus, also not suitable for on-line applications.

To realize effective and fast hot spot temperature calculations, the potential hot spot temperatures were determined by the average temperature of the hot spot voxels with the largest heating potential. The heating potential of a voxel is represented by the maximum eigenvalue of its temperature matrix [34]. The 1 cm outer rim of the patient was excluded in this process, since although these voxels have a large heating potential, no temperature hot spots will occur in the most superficial layers due to strong bolus cooling. The percentage of voxels with the largest heating potential can be selected by the user. In the present study, percentages of 1, 10, 50 and 100% were evaluated.

In case of a treatment limiting hot spot, the re-optimization of phase-amplitude settings was performed with a hard constraint to suppress the indicated hot spot. The real hot spot temperature was assumed to be 45 °C, since that is the level at which a pain sensation is experienced, and which can lead to irreversible tissue damage after too long exposure [15]. In a previous clinical pilot study, on-line adaptive treatment planning was applied to suppress hot spots, and in those patient cases the mean and median hot spot temperature reduction was ~1 °C [40]; also assuming the real hot spot temperature to be 45 °C. Therefore, in this study, a constraint was set to the treatment limiting hot spot location to realize a temperature reduction of 1 °C, after re-scaling the hot spot temperature to 45 °C. To avoid introducing new hot spots, hard constraints were set to all other potential hot spot locations, such that the current temperature level will increase at most 0.5 °C. Furthermore, power constraints were applied to the waveguides to avoid clinically unrealistic amplitude settings. A waveguide should at least deliver 10% of the total power, although not more than 40%. The total applied power should remain constant.

#### 2.3.2. Objective Functions

The aim in clinical hyperthermia is always to maximize the tumor temperature, and T90, i.e., the temperature at least achieved in 90% of the tumor volume is an important clinical parameter. Therefore, the objective functions used maximized T90. Hard constraints were applied to avoid a large temperature increase at other potential hot spot locations; to further minimize this temperature increase, also the use of additional penalty terms was evaluated. Penalty terms were defined for either the maximum increase in all potential hot spot temperatures, or the sum of the increase in potential hot spot temperatures. This yields the following three optimization goals:

(3) $$\max\left(T_{90}-\underset{\begin{array}{c} potential\\ hot\;spots \end{array}}{\max}\max\left(0,\;\left(T_{new}-T_{old}\right)\right)\right)$$

(4) $$\max\left(T_{90}-\sum_{\begin{array}{c} potential\\ hot\;spots \end{array}}\;\max\left(0,\;\left(T_{new}-T_{old}\right)\right)\right)$$

(5) $$\max\left(T_{90}\right)\;$$

all subject to hard normal tissue and antenna constraints, as described in Section 2.3.1. *T_old_* and *T_new_* represent the hot spot temperature before and after the re-optimization, respectively.

### 2.4. Patient and Event Selection

For evaluation of the re-optimization algorithms, we used the patient group from Kok et al. (2018) [39], where SAR/temperature changes after phase-amplitude steering were evaluated for patients with pelvic tumors to determine the correlation between measurements and simulations. All these patients received the hyperthermia treatment according to our standard clinical protocol, with protocol/experience-based steering. We selected steering events that were registered as action to suppress treatment limiting hot spots. Bladder cancer patients were excluded for this study, since the correlation between measured and simulated SAR/temperature changes in the bladder was rather weak (due to convection in the bladder fluid, not accounted for in standard treatment planning), and the target temperature is an optimization/evaluation parameter. This selection resulted in 22 phase-amplitude steering events in 16 locally advanced cervical cancer patients (mean age 67 ± 13 y). The average tumor size was 111 ± 69 cc. The average fat percentage in the patient models was 51.4 ± 9.4%; for muscle and bone these percentages were 42.2 ± 8.8% and 4.7 ± 0.7%, respectively. The registered hot spot locations per event and patients are listed in Table 2; in some cases two hot spots occurred simultaneously.

### 2.5. Evaluation

For each hot spot event in Table 2, re-optimization of phase-amplitude settings was performed to suppress the hot spot (or hot spots). To assess clinical feasibility for on-line use the re-optimization time was evaluated for each of the different strategies (i.e., objective Equations (3)–(5) and maximum eigenvalue percentages of 1, 10, 50 and 100%). To evaluate effectiveness in hot spot suppression, all voxels in the treatment limiting hot spot region were evaluated and the predicted maximum temperature reduction was determined. To evaluate the risk of newly induced hot spots, the overall maximum increase in the other potential hot spot regions and the change in predicted overall maximum in the whole patient volume were determined. For each re-optimization strategy, these predictions were compared with predictions for the clinically applied protocol/experience-based phase-amplitude adjustments.

Regarding the target temperatures, the predicted T90 and the temperature changes along the thermometry probes were evaluated. To reconstruct the simulated temperature along the thermometry probe trajectories in the cervix, track paths were delineated from the CT scan using the Plan2Heat module jTracktool [45]. Using these track paths the temperature was extracted from the simulated distributions with a 5 mm interval (i.e., the sensor spacing of the thermocouple probes) along the track. Spatial average values were calculated and simulated average temperatures were scaled per location with a factor such that the simulated value before changing the antenna settings corresponds to the measurement value. Next, the change in temperature due to adapting the antenna settings was determined. For each re-optimization strategy, we compared these predicted temperature changes along the probe with predictions for the clinically applied phase-amplitude adjustments, as well as with the real measurements.

All evaluations were first performed for a potential hot spot region diameter of 5 cm. For the most effective strategy, the influence of the hot spot diameter was also evaluated, comparing diameters of 2.5, 5, 7.5 and 10 cm.

## 3. Results

**Figure 2:**
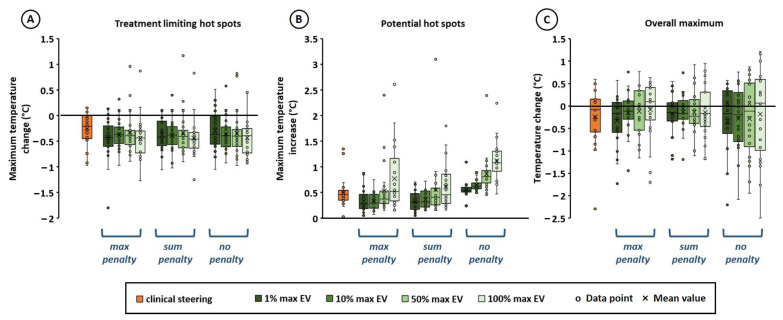
Effect of different re-optimization strategies on the predicted temperature at treatment limiting hot spot locations (**A**), other potential hot spot locations (**B**) and the overall maximum temperature (**C**). Results are compared to simulations for the clinically applied protocol/experience-based steering strategy during treatment. Max penalty, sum penalty and no penalty refer to the re-optimization goal functions in Equations (3), (4) and (5), respectively. To efficiently calculate a hot spot temperature during optimization, the average temperature of the voxels with the x% largest heating potential was calculated, which are the voxels with the largest maximum eigenvalues (EV) of their temperature matrix (*T* in Equation (2). Values of x of 1, 10, 50 and 100% were evaluated. The hot spot diameter was 5 cm.

The effect of different re-optimization strategies on treatment limiting hot spot suppression as well as the maximum temperature increase at other potential hot spot locations and the overall normal tissue temperature maximum, are shown in Figure 2. The hot spot diameter was 5 cm. The effect of the clinically applied steering strategy by experienced treatment operators, is also shown. We observed that effective treatment limiting hot spot suppression was predicted for all strategies, which is, with exception of a few outliers, equally effective as the clinical strategy (Figure 2A). The difference in predicted mean hot spot temperature change compared to the clinical strategy was less than 0.2 °C for all strategies, and the standard deviation differed less than 0.1 °C. Considering the individual difference in predicted hot spot reductions between planning-based and clinical strategies for all 22 hot spots and steering actions, we see that the overall mean difference is −0.1–−0.2 °C (±0.3–0.4 °C). This indicates a mild trend that planning-based steering yields a slightly larger reduction in hot spot temperature. However, since real and predicted temperature changes might deviate ±0.1 °C [39] and small differences might not be perceptible by the patient, this difference is not considered clinically relevant and planning-based and clinical strategies are considered equally effective. Although hard constraints were applied to other potential hot spot locations to avoid inducing new hot spots, adding a penalty term was an effective strategy to further avoid a large temperature increase at other locations (Figure 2B,C). Both penalties in goal function (3) and (4) were effective. As expected, the risk of insufficiently suppressing the treatment limiting hot spot and/or inducing new hot spots increased when a larger number of voxels was included to determine the average hot spot temperature. In case voxels with the 50% or 100% largest heating potential were used (50% max EV and 100% max EV), this averaging often underestimates the hot spot temperature and a higher temperature increase at other potential hot spot locations was observed and the risk of outliers increased (Figure 2A,B). These outliers represent the limited number of cases where the average temperature estimating the hot spot temperature in the model, deviated largely from the real maximum temperature in the hot spot region. The fact that there are few outliers with a large temperature increase indicates that this averaging strategy to realize fast calculations works well, especially when only those voxels with the largest heating potential are included (i.e., 1% and 10% max EV). In those cases, and when including penalty terms, the average difference in predicted maximum potential hot spot increase and overall maximum temperature change compared to the clinical strategy was less than 0.15 °C (and with a comparable spread), which is not a clinically relevant difference and thus the strategies can be considered equally effective.

**Figure 3:**
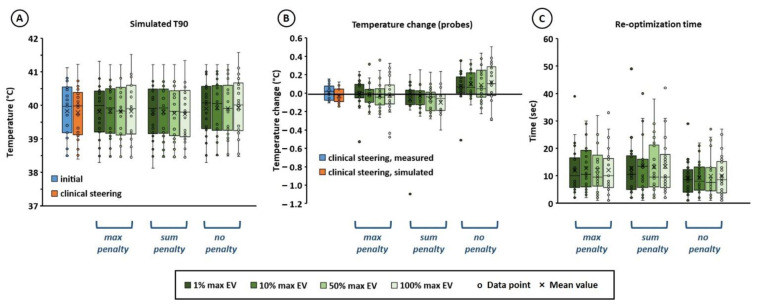
Effect of different re-optimization strategies on the simulated T90 (**A**) and the temperature change along the thermometry probes (**B**). The re-optimization calculation time per strategy is indicated in (**C**) for an Intel Xeon^®^ E5-1650 v3 3.5 GHz running CentOS 6.8. Results are compared to simulations for the clinically applied protocol/experience-based steering strategy during treatment (**A**,**B**), as well as to probe measurements (**B**). Max penalty, sum penalty and no penalty refer to the re-optimization goal functions in Equations (3), (4) and (5), respectively. To efficiently calculate a hot spot temperature during optimization, the average temperature of the voxels with the x% largest heating potential was calculated, which are the voxels with the largest maximum eigenvalues (EV) of their temperature matrix (*T* in Equation (2). Values of x of 1, 10, 50 and 100% were evaluated. The hot spot diameter was 5 cm.

Figure 3A shows that for all re-optimization strategies, the simulated target T90 after re-optimization was quite comparable to the initial value before re-optimization, and also quite similar to the simulated T90 for the clinically applied steering strategy based on experience. Both mean and standard deviation were equal within 0.1 °C, which is not a clinically relevant difference. Considering the individual absolute difference in predicted T90 between planning-based and clinical strategies for all 22 hot spots and steering actions, we see that the mean absolute difference is also very low, i.e., 0.1 ± 0.1 °C when applying a penalty term and 1% or 10% max EV. When evaluating the change in temperature along the thermometry probes (Figure 3B), it was observed that this was approximately 0 °C for the clinically applied strategy, both simulated and measured during treatment. For both optimization goal functions using a penalty term, the predicted temperature change along the probes was comparable to the clinical strategy. When only re-optimizing T90, without a penalty term, a slight increase of typically 0.1–0.2 °C is observed. However, this is not considered clinically relevant and as indicated in Figure 2B,C, this also yields a risk of inducing new hot spots at other locations. The re-optimization time was quite similar for all re-optimization strategies (Figure 3C). Re-optimization of the phase-amplitude settings took typically ~10 s (mean ± std = 12 ± 9 s) on an Intel Xeon^®^ E5-1650 v3 3.5 GHz running CentOS 6.8, and always less than 1 min, which is sufficiently fast for on-line application. In about 3% of the cases (i.e., 9 out of the overall total of 22 × 4 × 3 = 264 re-optimizations), no feasible solution satisfying all constraints was found. As a back-up solution for these situations, a form of standard steering was implemented. The power of the waveguide closest to the selected treatment limiting hot spot location was then reduced by 15%, which was redistributed over the other waveguides to maintain a fixed power level; e.g., in case of hot spot 29, pubic bone, the power supplied by the top waveguide is reduced by 15%. This is quite similar to commonly applied clinical steering strategies.

**Figure 4:**
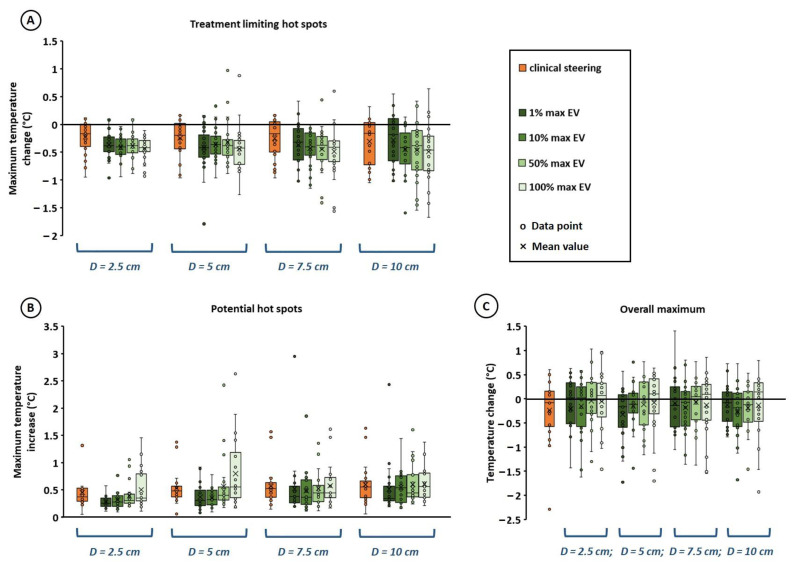
Effect of potential hot spot diameter (*D* = 2.5, 5, 7.5 or 10 cm) on the predicted temperature at treatment limiting hot spot locations (**A**), other potential hot spot locations (**B**) and the overall maximum temperature (**C**), when using a re-optimization goal function with a penalty term for the maximum increase of all potential hot spot temperatures (Equation (3). Results are compared to simulations for the clinically applied protocol/experience-based steering strategy during treatment. To efficiently calculate a hot spot temperature during optimization, the average temperature of the voxels with the x% largest heating potential was calculated, which are the voxels with the largest maximum eigenvalues (EV) of their temperature matrix (*T* in Equation (2). Values of x of 1, 10, 50 and 100% were evaluated.

Thus, based on the results summarized in Figure 2 and Figure 3, an objective function including a penalty term would be most effective. Next, the influence of the hot spot diameter was evaluated for the most effective strategy. Both a penalty term for the maximum temperature increase in all potential hot spots (Equation (3)), and the sum of the increase in potential hot spot temperatures (Equation (4)) were effective, with no clearly pronounced difference. However, Equation (3) was selected for further evaluation, since Figure 3B shows a weak trend that using Equation (4), temperatures along the thermometry probe decrease slightly, and since the risk of not finding a solution was slightly lower in case of Equation (3). The potential hot spot diameter was varied between 2.5, 5, 7.5, and 10 cm and Figure 4 shows the effect of different re-optimization strategies on treatment limiting hot spot suppression as well as the maximum temperature increase at other potential hot spot locations and the overall normal tissue temperature maximum. Again, it was observed that the risk of insufficiently suppressing the treatment limiting hot spot and/or inducing new hot spots increased when a larger number of voxels was included to determine the average hot spot temperature (50% max EV and 100% max EV). No pronounced effect of the diameter was observed. When using 1% max EV or 10% max EV, the average predicted decrease in treatment limiting hot spot temperature deviated less than 0.2 °C from the clinical steering strategy, and the standard deviation differed less than 0.1 °C, for all hot spot diameters. Similarly, the average maximum increase in other potential hot spot locations deviated less than 0.2 °C from the clinical steering strategy, and the standard deviation differed less than 0.15 °C. The overall mean maximum temperature change deviated less than 0.1 °C from the clinical steering strategy, and the standard deviation differed less than 0.25 °C. As observed also in Figure 2, there is a mild trend that planning-based steering yields a slightly larger reduction in hot spot temperature. However, since real and predicted temperature reductions might deviate ±0.1 °C [39] and small differences might not be perceptible by the patient, these small differences are not considered clinically relevant and thus the strategies can be considered equally effective.

**Figure 5:**
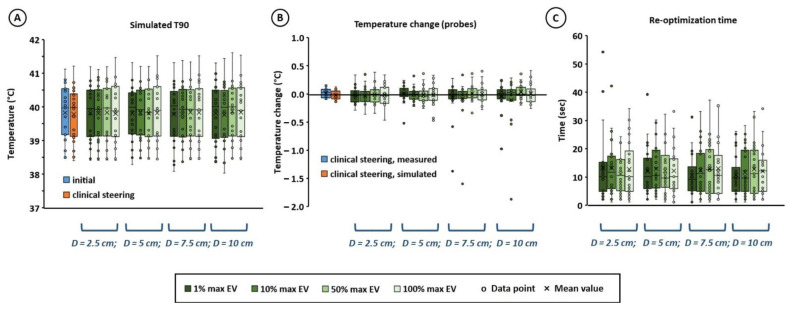
Effect of potential hot spot diameter (*D* = 2.5, 5, 7.5 or 10 cm) on the simulated T90 (**A**) and on the temperature change along the thermometry probes (**B**), when using a re-optimization goal function with a penalty term for the maximum increase in all potential hot spot temperatures (Equation (3)). The re-optimization calculation time is indicated in (**C**) for an Intel Xeon^®^ E5-1650 v3 3.5 GHz running CentOS 6.8. Results are compared to simulations for the clinically applied protocol/experience-based steering strategy during treatment (**A**,**B**), as well as to probe measurements (**B**). To efficiently calculate a hot spot temperature during optimization, the average temperature of the voxels with the x% largest heating potential was calculated, which are the voxels with the largest maximum eigenvalues (EV) of their temperature matrix (*T* in Equation (2). Values of x of 1, 10, 50 and 100% were evaluated.

Figure 5 shows the effect of the potential hot spot diameter on the simulated T90 and the temperature change along the thermometry probes. For all diameters, the simulated target T90 after re-optimization was quite comparable to the initial value before re-optimization, and also quite similar to the simulated T90 for the clinically applied steering strategy based on experience (Figure 5A). Both mean and standard deviation were equal within 0.1 °C, which is not a clinically relevant difference. Considering the individual absolute difference in predicted T90 between planning-based and clinical strategies for all 22 hot spots and steering actions, we see that the mean absolute difference is again also very low, i.e., typically 0.1 ± 0.2 °C. When evaluating the temperature along the thermometry probes (Figure 5B), it was observed that this temperature remains quite constant for any diameter, and similar to the clinically applied strategy. The mean temperature change was less than 0.1 °C. No pronounced influence of the diameter was observed, although the risk of outliers slightly increased for a larger potential hot spot region diameter, since a larger number of voxels is included in the averaging to estimate the hot spot temperature. As expected, the hot spot diameter also had no influence on re-optimization calculation times (Figure 5C).

**Figure 6:**
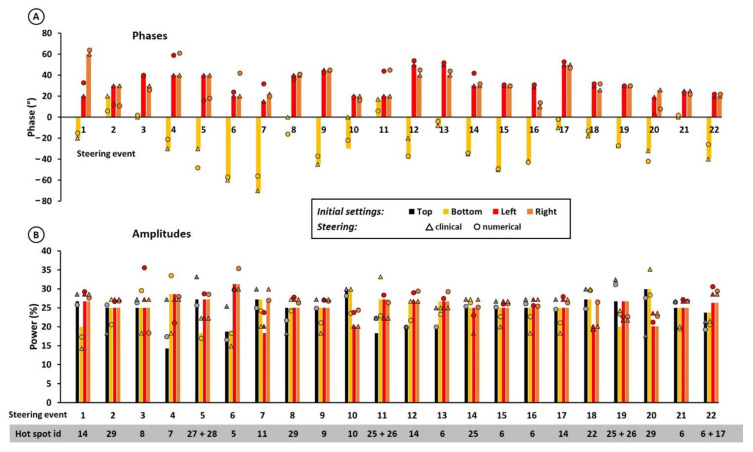
Clinically applied protocol/experience-based phase (**A**) and amplitude (**B**) adjustments and numerically re-optimized settings for a potential hot spot diameter of 5 cm, using a re-optimization goal function with a penalty term for the maximum increase in all potential hot spot temperatures (Equation (3)) and using voxels with the 10% maximum heating potential to calculate the hot spot temperature (10% max EV). The top antenna was always used as a reference, i.e., phase 0°. Hot spot id refers to the anatomical locations in Figure 1A.

Similarities were observed in the steering strategies, when comparing clinically applied phase-amplitude settings with numerically re-optimized settings. Clinical steering for hot spot suppression predominantly uses amplitude steering while keeping phases quite constant to maintain a focus at the target location. Numerically achieved re-optimized settings generally also prescribed amplitude steering. For amplitude steering, a similarity in steering direction was also observed in many cases, i.e., when an amplitude was increased or decreased in clinical steering, numerical optimization also resulted in increase/decrease, albeit with different amplitudes. Figure 6 shows clinically applied phase-amplitude adjustments and numerically re-optimized settings for a potential hot spot diameter of 5 cm, using a re-optimization goal function with a penalty term for the maximum increase in all potential hot spot temperatures (Equation (3)) and using voxels with the 10% maximum heating potential to calculate the hot spot temperature (10% max EV).

## 4. Discussion

In this paper, we presented fast and advanced temperature-based strategies for adaptive on-line re-optimization of phase-amplitude settings, to overcome treatment limiting hot spots during locoregional hyperthermia treatments. Considering target and hot spot temperatures, results were compared to predictions and tumor temperature measurements for clinically applied protocol/experience-based steering strategies. A similar effectiveness to clinical experience was predicted. This is a very promising result in view of there being about 30 years of experience with locoregional hyperthermia and experience-based steering at our department. When numerical algorithms can match such long-term experience, the overall treatment quality in hyperthermia centers can significantly improve. The results also imply that treatments become less dependent on the experience of the center/operator by implementing these planning-based strategies.

Although temperature-based optimization is computationally more expensive than the commonly applied SAR-based optimization, we managed to achieve average re-optimization times of about 10 s. This was realized by efficient superposition to calculate steady-state temperatures (Equation (2)) and limiting the number of normal tissue constraints to only the potential hot spot regions, with their temperature represented by the average temperature of the voxels with the largest heating potential. Superposition was possible due to the use of constant enhanced perfusion levels. In reality, perfusion is temperature-dependent and thermal models including temperature-dependent perfusion have been proposed [51,53]. Ignoring the temperature-dependency of perfusion can result in under/overestimation of absolute temperatures levels [36,37]. However, the use of temperature-dependent perfusion values will not necessarily improve the quality of adaptive treatment planning as proposed in this study, since changes in simulated temperatures are used as a predictor, rather than the absolute values. Furthermore, including temperature-dependent perfusion values does not allow fast superposition to calculate temperatures, which makes calculations much more computationally expensive and not feasible for on-line re-optimization.

Our clinical steering strategy mainly applies amplitude adjustments (Figure 6). Amplitude adjustments are a preferred and effective strategy adopted in empirical steering guidelines by our and other experienced centers [54]. This makes sense as phase settings mainly determine the location of the heating focus, so avoiding significant phase changes and applying amplitude steering ensures maintaining the focus at the target location, as optimized at the start of treatment. Furthermore, the location of treatment limiting hot spots is usually relatively superficial, and amplitude adjustments directly affect the local temperature close to the antenna. We observed that numerical re-optimization also identified this principle as an optimal solution to suppress hot spots while maintaining optimal target heating; numerical re-optimization also predominantly prescribed amplitude steering.

Based on the positive results of this simulation study, a next step would be clinical evaluation of temperature-based adaptive re-optimization and comparing this with clinical experience. A cross-over trial by Franckena et al. comparing SAR-based treatment planning-guided steering with experience-based steering demonstrated the feasibility of treatment planning-based steering [43]. In the first half of a patient’s treatment series, similar target temperatures were achieved with both planning-guided and empirical steering. However, in the second half of the treatment series, planning-guided steering resulted in significantly lower (~0.5 °C) target temperatures, indicating the necessity to further improve treatment planning-based steering. Several (technical and patient) factors could explain this decreasing reliability and require further research, such as planning strategy, decreasing patient tolerance and a changing anatomy over the treatment course, while the planning is based on anatomical information of the initial planning CT scan.

The planning strategy applied in the trial of Franckena et al. was a SAR-based re-optimization, developed as an add-on to Sigma HyperPlan, and using adjusted weight factors to reduce the SAR in treatment limiting hot spot regions [42,43]. Although there is some correlation between SAR and temperature, the local temperature determines whether a hot spot becomes treatment limiting or not. The temperature at a potential hot spot location depends on SAR. However, it also depends on thermal processes, such as thermal conduction, blood perfusion and bolus cooling, which are not accounted for in SAR-based (re-)optimization. Furthermore, SAR-based optimization is less intuitive for the operator and it is difficult to determine how much SAR reduction is required to resolve a hot spot complaint. This might partly explain the challenges Franckena et al. encountered in solving hot spot-related complaints. Our temperature-based approach accounts for relevant thermal processes and is more practical and intuitive for clinical use. Moreover, a unique approach is that our strategy not only aims to reduce the temperature at treatment limiting hot spot locations, but also to prevent inadvertently inducing new hot spots at other potential hot spot locations while adjusting the phase-amplitude settings to suppress the original hot spot. This feature would make it more robust for clinical applications compared to existing SAR-based strategies.

Another important aspect of re-optimization algorithms is robustness in terms of specification of potential hot spot locations. Since the operating frequency of the ALBA-4D system is 70 MHz, the wavelength in muscle tissue is approximately 50 cm. This means that true small scale steering is not possible and the re-optimization results will not be very sensitive to the exact specified position of the potential hot spots [55]. This is confirmed by the observation that the potential hot spot region diameter did not significantly affect re-optimization results (Figure 4 and Figure 5). The fact that the diameter is not an important factor implies that results are also not strongly dependent on the exact potential hot spot location indicated by the user. Since all locoregional heating systems operate in the frequency range between 60 and 120 MHz [20], this will probably also hold when combining this temperature-based optimization strategy with other locoregional devices. Results of the present study can certainly be extrapolated to the widely used BSD Sigma-60 system, with four antenna pairs and similar heating characteristics as the ALBA-4D system [56]. With an increased number of antennas and thus an increased number of degrees of freedom, e.g., for the BSD Sigma-Eye system, the exact hot spot location could be slightly more important, requiring further investigation. However, re-optimization is still expected to be quite robust to potential hot spot location because of the large wavelengths used in locoregional heating systems.

The relatively large wavelength of locoregional heating systems can also explain why no improvement in tumor temperature was predicted compared to experience-based steering. This is in line with simulation study results of Canters et al., who evaluated the possible gain in tumor temperatures by optimization for the BSD Sigma-60 system [55]. They showed that the potential of treatment planning to optimize the SAR distribution is limited [55]. The maximum improvement was in the order of 5%, which would lead to a temperature increase of about 0.2 °C [43,55]. Although the performance of planning-based re-optimization is expected to improve using temperature-based strategies, the number of degrees of freedom is relatively small with four antennas or antenna pairs and a wavelength in the order of 50 cm, so the steering possibilities remain a limitation. Using heating systems with higher frequencies and/or more antennas, such as the AMC-8 system and the BSD Sigma-Eye system, more flexible steering and thus a tumor temperature increase is expected to be possible [57,58]. However, clinical use of these systems showed that realizing improved tumor heating by exploiting the increased steering possibilities remains challenging [59]. Thus, investigation of advanced (adaptive) treatment planning tools to fully exploit the benefits of such systems enabling 3D steering is worthwhile [59,60].

Evaluation of the benefit of using temperature-based planning during hyperthermia treatment is a subject of ongoing research within our department. After positive results in pilot studies with treatment planning and planning-assisted manual re-optimization [40,41], we will continue to investigate the benefit of automatic re-optimization as proposed in this paper. To ensure optimal patient safety, operators will always have to check the re-optimized phase-amplitude settings and manually apply them to the heating system. Furthermore, continuous monitoring of the thermometry probe registrations and patient feedback, as part of our standard treatment protocol, remains important. When successful, treatment planning-based steering would match empirical steering by very experienced operators, realizing a constant operator-independent heating quality. Subsequently, effective treatment limiting hot spot suppression and absence of new hot spots could allow a total power increase to realize a better heating quality.

## 5. Conclusions

Fast and advanced temperature-based strategies for adaptive on-line re-optimization of phase-amplitude settings were presented to suppress treatment limiting hot spots which may occur during locoregional hyperthermia treatments. An effectiveness similar to re-optimization based on long-term clinical experience was predicted. A major advantage is that treatments would become less dependent on the experience of the hyperthermia center/operator, thereby improving the overall treatment quality in hyperthermia centers. Further clinical evaluation is a subject of ongoing research.

## Figures and Tables

**Table 1:** Values of the dielectric and thermal tissue properties at 70 MHz used in the simulations.

Tissue	*σ* (S m^−1^)	*ε_r_* (-)	*ρ* (kg m^−3^)	*c* (J kg^−1^ °C^−1^)	*k* (W m^−1^ °C ^−1^)	*W_b_* (kg m^−3^ s^−1^)
Air	0	1	1.29	1000	0.024	0
Bone	0.05	10	1595	1420	0.65	0.12
Fat	0.06	10	888	2387	0.22	1.1
Muscle	0.75	75	1050	3639	0.56	3.6
Tumor	0.74	65	1050	3639	0.56	1.8

electrical conductivity (*σ* [S m^−1^]); relative permittivity (*ε_r_* [-]); density (*ρ* [kg m^−3^]); specific heat capacity (*c* [J kg^−1^ °C^−1^]); thermal conductivity (*k* [W m^−1^ °C^−1^]) and perfusion (*W_b_* [kg m^−3^ s^−1^]). NB: perfusion values are enhanced to account for a physiological response to a temperature rise in the hyperthermic range [48,51].

**Table 2:** Registered treatment limiting hot spot locations for the patients and events included in this study. Anatomical location of hot spot location identifiers is shown in Figure 1A.

Event	Patient	Hot Spot Identifier
1	1	14
2	2	29
3	2	8
4	3	7
5	3	27 + 28
6	4	5
7	4	11
8	5	29
9	6	9
10	7	10
11	7	25 + 26
12	8	14
13	8	6
14	9	25
15	10	6
16	10	6
17	11	14
18	12	22
19	13	25 + 26
20	14	29
21	15	6
22	16	6 + 17

## Data Availability

The data presented in this study are available on reasonable request. The data are not publicly available due to privacy.
